# Culture-dependent and -independent methods revealed an abundant myxobacterial community shaped by other bacteria and pH in Dinghushan acidic soils

**DOI:** 10.1371/journal.pone.0238769

**Published:** 2020-09-14

**Authors:** Chunling Wang, Yingying Lv, Anzhang Li, Qing Yao, Guangda Feng, Honghui Zhu

**Affiliations:** 1 State Key Laboratory of Applied Microbiology Southern China, Guangdong Provincial Key Laboratory of Microbial Culture Collection and Application, Guangdong Open Laboratory of Applied Microbiology, Guangdong Microbial Culture Collection Center (GDMCC), Guangdong Institute of Microbiology, Guangdong Academy of Sciences, Guangzhou, Guangdong, China; 2 College of Horticulture, South China Agricultural University, Guangzhou, Guangdong, China; Universidade de Coimbra, PORTUGAL

## Abstract

Myxobacteria are one of the most promising secondary metabolites producers. However, they are difficult to isolate and cultivate. To obtain more myxobacteria and know the effects of environmental factors on myxobacterial community, we characterized myxobacterial communities in Dinghushan acidic forest soils of pH 3.6–4.5 with culture-dependent and -independent techniques, and analyzed environmental factors shaping myxobacterial communities. A total of 21 myxobacteria were isolated using standard cultivation methods, including eleven isolates of *Corallococcus*, nine isolates of *Myxococcus* and one isolate of *Archangium*, and contained three potential novel species. In addition, a total of 67 unknown myxobacterial operational taxonomic units (OTUs) were obtained using high-throughput sequencing method. The abundance of *Myxococcales* account for 0.9–2.2% of bacterial communities, and *Sorangium* is the most abundant genus (60.1%) in *Myxococcales*. Correlation analysis demonstrated that bacterial diversity and soil pH are the key factors shaping myxobacterial community. These results revealed an abundant myxobacterial community which is shaped by other bacteria and pH in Dinghushan acidic forest soils.

## Introduction

Myxobacteria are ubiquitous gram-negative rod-shaped bacteria which are distributed all over the world, even including extreme environments [1, 2]. Because of their unique social lifestyle, myxobacteria are of particular interest in the study of multicellular behaviors of bacteria [3, 4]. During vegetative growth, myxobacterial rod-shaped cells move by gliding motility and feed on a broad range of bacteria and fungi [2]. Upon starvation, vegetative cells aggregate into fruiting bodies forming resistant myxospores which can be dormant for several years [5]. Myxobacteria are able to produce prospective novel secondary metabolites for pharmaceutical research. More than 100 basic core metabolites originating from myxobacteria have been identified, most of which have antibiotic, anti-malarial, immunosuppressive, antiviral or insecticidal activities [6]. However, myxobacteria are difficult to isolate and cultivate, with only 74 described species to date [2, 7, 8]. According to Mohr [2], more than 99% myxobacteria are uncultured and uncharacterized due to the lack of optimum isolation methods.

The deep insights into the myxobacterial communities in different habitats and the environmental factors shaping the communities maybe contribute to obtain these hidden groups. Previous studies demonstrated that myxobacteria accounted for 0.4–4.5% of all bacterial communities in different soil niches by analyzing 103 high-throughput sequencing data sets obtained from public databases and their abundance were correlated with site temperature, carbon-to-nitrogen ratio and pH values [9]. Myxobacteria preferred to survive in neutral or alkalescent habitats [2]. However, alkaline or acidic environments also seem to be suitable for myxobacteria [1]. Zhang *et al*. [10] isolated 58 salt-tolerant myxobacteria from saline-alkaline soils collected from Xinjiang, China and all these strains grew better with 1% NaCl. Mohr *et al*. [11] found an astonishing myxobacterial diversity in high moor and fen with pH between 4.0 and 7.0 with cultivation-based and cultivation-independent methods. Moreover, Mohr *et al*. [12] also investigated diversity of myxobacteria in two ecological habitats from Kiritmati Island and German compost. The results showed an overrepresentation of the genera *Myxococcus* spp. and *Corallococcus* spp. with standard cultivation and revealed a great potential of undescribed myxobacteria in both sampling sites.

Additionally, as social predators, myxobacteria exhibited a wide range of predation activities against both bacteria and fungi [13, 14], changing the structure and diversity of microbial community [15, 16]. Moreover, Wang *et al*. [17] latest reported that predatory *Myxococcales* are widely distributed in and closely correlated with the bacterial community structure of agricultural land. These pointed to the possibility that myxobacteria influence the communities of bacteria and fungi. Yet few studies analyze the importance of bacteria and fungi to myxobacterial community.

Dinghushan forest ecosystem contains a large number of microbial novel species and represents a hot spot area for studying the biodiversity of tropical and subtropical forest ecosystems [18, 19]. To obtain more myxobacterial sources, the intensive examination of neglected/extreme habitats and grasping the influence factors on myxobacterial community are necessary strategies. In this study, we investigated the communities of myxobacteia in Dinghushan forest soils, which is a neglected and extreme habitat, using culture-dependent and -independent (high-throughput Illumina sequencing) techniques. Additionally, the effects of biotic (bacterial diversity) and abiotic factors shaping myxobacterial community were analyzed and compared. We aimed to know the myxobacterial diversity and key factors to determine myxobacterial communities in Dinghushan acidic forest soils. These studies can add new information about myxobacterial diversity in subtropical acidic forest soils and guide to obtain more myxobacteria.

## Materials and methods

### Ethics statement

No specific permissions were required for the study, as it did not involve human or animals. Moreover, the sample sampling did not involve endangered or protected species.

### Soil samples collection

The soil samples were collected in September of 2017 from Dinghushan Biosphere Reserve (112°30′~112°33′E; 23°09′~23°11′N), which locates at Zhaoqing city in Guangdong province of China. It has a typical monsoonal climate with the annual average temperature of 22.3°C and rainfall of 1678 mm [18]. A total of 30 soil samples were collected from topsoil (5–15 cm) of 3 forest types (coniferous forest, coniferous-broadleaf mixed forest and monsoon evergreen broadleaf forest, 10 samples from each kind of forest) and immediately transported to laboratory in an ice box. Counterparts of 9 representative samples were drawn and stored at -80°C. Then, all 30 soil samples were air-dried and stored at 4°C.

### Isolation of myxobacteria with cultivation

**Fig 1:**
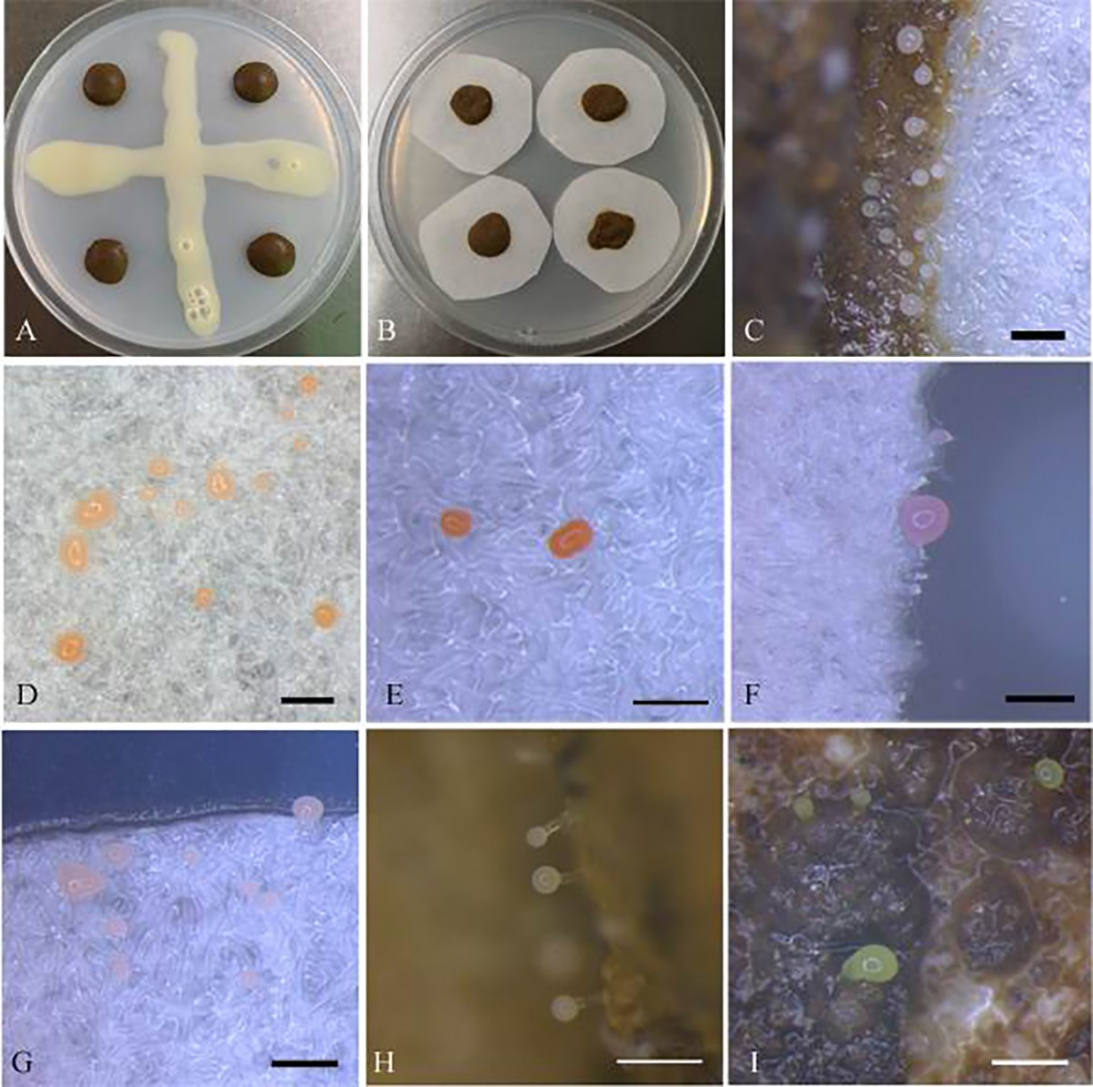
Isolation of myxobacteria using *E*. *coil* (A) and filter papers (B); Sectional fruiting bodies observed on filter papers (C-G) and on soil samples using *E*. *coli* as baits (H-I). Phase-contrast photographs of *M*. *stipitatus* K13C18031201, *M*. *stipitatus* 25S18041902, *M*. *stipitatus* Z6C18031901, *M*. *xanthus* K38C18041901, *Corallococcus* sp. K15C18031902, *Corallococcus* sp. K2CV092601 and *Corallococcus* sp. H22C18031201. Bars, C-E, G-I: 0.5 mm; F: 0.25 mm.

Myxobacteria were isolated using the standard techniques from the 30 air-dried samples: soil samples were soaked in actinomycin for 12 h to avoid fungal contamination, and then plated on WCX solid medium (CaCl_2_**·**2H_2_O 0.1%, cycloheximide 50 μg/mL, agar 1.5%, pH 7.2) with *E*. *coli* (Fig 1A) and filter papers as food sources (Fig 1B) [20]. Plates were incubated for 3–5 weeks at 30°C. Fruiting bodies were observed every three days using a stereoscope (Leica, M165C) and then transferred into VY/2 (Baker’s yeast 0.5%, CaCl_2_**·**2H_2_O 0.1%, Cyanocobalamin 0.5 mg/mL, agar 1.5%, pH 7.2) and MD1 agar (Casitone 0.3%, CaCl_2_**·**2H_2_O 0.07%, MgSO_4_**·**7H_2_O 0.2%, Cyanocobalamin 0.5 mg/mL, agar 1.5%, pH 7.2), respectively. Pure culture was obtained by continuous transfer of colony edge onto VY/2 or MD1 agar, and stored in 20% glycerol solution under -80°C.

### Phylogenetic analysis using 16S rRNA gene sequences of myxobacteria

The genomic DNA of pure culture was extracted using the improved Cetyltrimethylammonium Ammonium Bromide (CTAB) method [21] and 16S rRNA gene was sequenced by the universal primers 27F and 1492R [22]. Sequences obtained were compared with other 16S rDNA in Genbank (http://www.ncbi.nlm.nih.gov). A neighbor-joining tree [23] was constructed using MEGA 7.0 software based on the 16S rRNA gene sequences applying bootstrap values with 1000 replications. Evolutionary distance was calculated using Kimura’s two-parameter model [24]. The 16S rRNA gene sequences obtained had been submitted to the National Center for Biotechnology Information (NCBI) database under the accession numbers MK968786-MK968806.

### Genome sequencing and comparative genomic analysis

According to the phylogenetic analysis, 11 isolates in *Corallococcus* were divided into two groups, which represented different strains of two potential novel species. Because of the close 16S rRNA gene similarities of different strains, we selected strains H22C18031201 and Z5C101001 as two representative species, and their draft genomes were sequenced on the Illumina Hiseq platform using a 2×150 bp paired-end reads at Shanghai Personal Biotechnology Co., Ltd. (Personalbio, Shanghai). Sequencing reads were assembled into contigs and scaffolds by using A5-miseq v20150522 [25]. The genome sequences of H22C18031201 and Z5C101001 had been submitted to NCBI database under the accession number QNUN00000000 and VKLU00000000, respectively.

The genome sequences of *C*. *coralloides* DSM 2259^T^ (CP003389), *M*. *fulvus* DSM 16525^T^ (FOIB00000000), *M*. *xanthus* DSM 16526^T^ (FNOH00000000), *M*. *virescens* DSM 2260^T^ (FNAJ00000000), *M*. *stipitatus* DSM14675^T^ (CP004025), *M*. *macrosporus* DSM 14697^T^ (CP022203), *Archangium gephyra* (CP011509) and *Archangium* sp. Cb G35 (MPOI01000017) were downloaded from NCBI database for the comparative study. The digital estimates for DNA-DNA hybridization (dDDH) values were calculated between the genome dataset pairs by using website at https://ggdc.dsmz.de/ provided on the Genome-to-Genome Distance Calculator GGDC [26]. Average nucleotide identity (ANI) calculator was performed by using the OrthANIu algorithm (https://www.ezbiocloud.net/tools/ani) [27].

### Soil physicochemical properties

The 9 counterparts of soil samples for high-throughput sequencing were used to measure physicochemical properties. Soil pH was measured with deionized water (2.5:1, w/v) using a glass electrode (Sartorius PB-10). The contents of organic matter (OM) were determined by titration after wet oxidation with H_2_SO_4_ and K_2_Cr_2_O_7_ [28]. Available nitrogen (AN), available phosphorus (AP) and available potassium (AK) contents were analyzed with alkali-hydrolyzed reduction diffusing method [29], colorimetric method (UV754N, YOKE INSTRUMENT) [30] and flame photometer method (FP6410, SH1717 China) [31], respectively. Ammonium (NH4^+^) and nitrate (NO3^-^) contents were analyzed according to the described method by Liu *et al*. using UV spectrophotometry and Nessler’s reagent colorimetry, respectively [32]. Cation exchange capacity (CEC) were determined using the ferric ammonium EDTA method (KDN-103A) [33].

### DNA extraction and the high-throughput sequencing

Genomic DNA was extracted from the 9 soil samples stored at -80°C using the PowerSoil^®^ DNA isolation kit (MoBio Laboratories Inc.) according to the manufacturer’s instructions. The DNA quality was tested with 1% agarose gel electrophoresis and DNA concentration was determined with a Nanodrop 2000C Spectrophotometer.

Sequencing was performed on the Illumina PE300 platform at Shanghai Majorbio Bio-pharm Technology Co., Ltd. The V3-V4 region of 16S rDNA was amplified with the universal primers 338F (ACTCCTACGGGAGGCAGCAG) and 806R (GGACTACHVGGGTWTCTAAT) as previously described [34] that contained the Illumina adapter overhang nucleotide sequences. The raw sequence reads were de-multiplexed, quality-filtered, processed and analyzed using QIIME [35]. The raw sequence data had been deposited in the Sequence Read Archive at NCBI dababase (accession number SRP9637593-SRP9637601).

### Bioinformatics data analysis

Sequences with ≥97% similarity were assigned to the same operational taxonomic unit (OTU) by UCLUST clustering [36]. OTU tables from all bacteria and myxobacteria were output for further analysis. The Chao index was calculated with QIIME to estimate the richness of bacterial communities. The Shannon index was calculated diversity of bacterial communities in QIIME. The correlations between environmental factors and myxobacterial alpha-diversity were revealed by Spearman analysis. The relationships between environmental factors and myxobacterial community structure were calculated with Mantel test based on pairwise Bray-Curtis distance matrix. Histograms of myxobacterial communities based on the high-throughput sequencing were constructed using Graphpad prism 7.0 software (Graphpad Software, San Diego, CA, USA). The network analysis was performed with a random matrix theory (RMT)-as default setup on an online pipeline (http://ieg4.rccc.ou.edu/MENA) and visualized using Cytoscape software [37].

## Results

### Cultivation of myxobacteria from Dinghushan forest soils

A total of 66 myxobacterial strains from 30 soil samples were isolated. Based on 16S rRNA gene sequences and morphological characteristics, potential duplicates were excluded from further analyses. Finally, a total of 21 isolates were further analysed. Sectional fruiting body pictures were shown in Fig 1C–1I. Most of these fruiting bodies were spherical or ellipsoid in shape, and white, yellow, orange red or pale pink. In addition, during the myxobacterial isolation, 4 novel strains of *Chitinophaga varians* 10-7W-9003^T^ [38], *Chitinophaga flava* K3CV102501^T^ [39], *Chitinophaga* sp. K2CV101002 (unpublished) and *Chitinophaga silvisoli* K20C18050901^T^ [40], and 1 strain of *Deminuibacter soli* K23C18032701^T^ [41], belonging to the family *Chitinophagaceae*, were isolated as myxobacteria.

**Fig 2:**
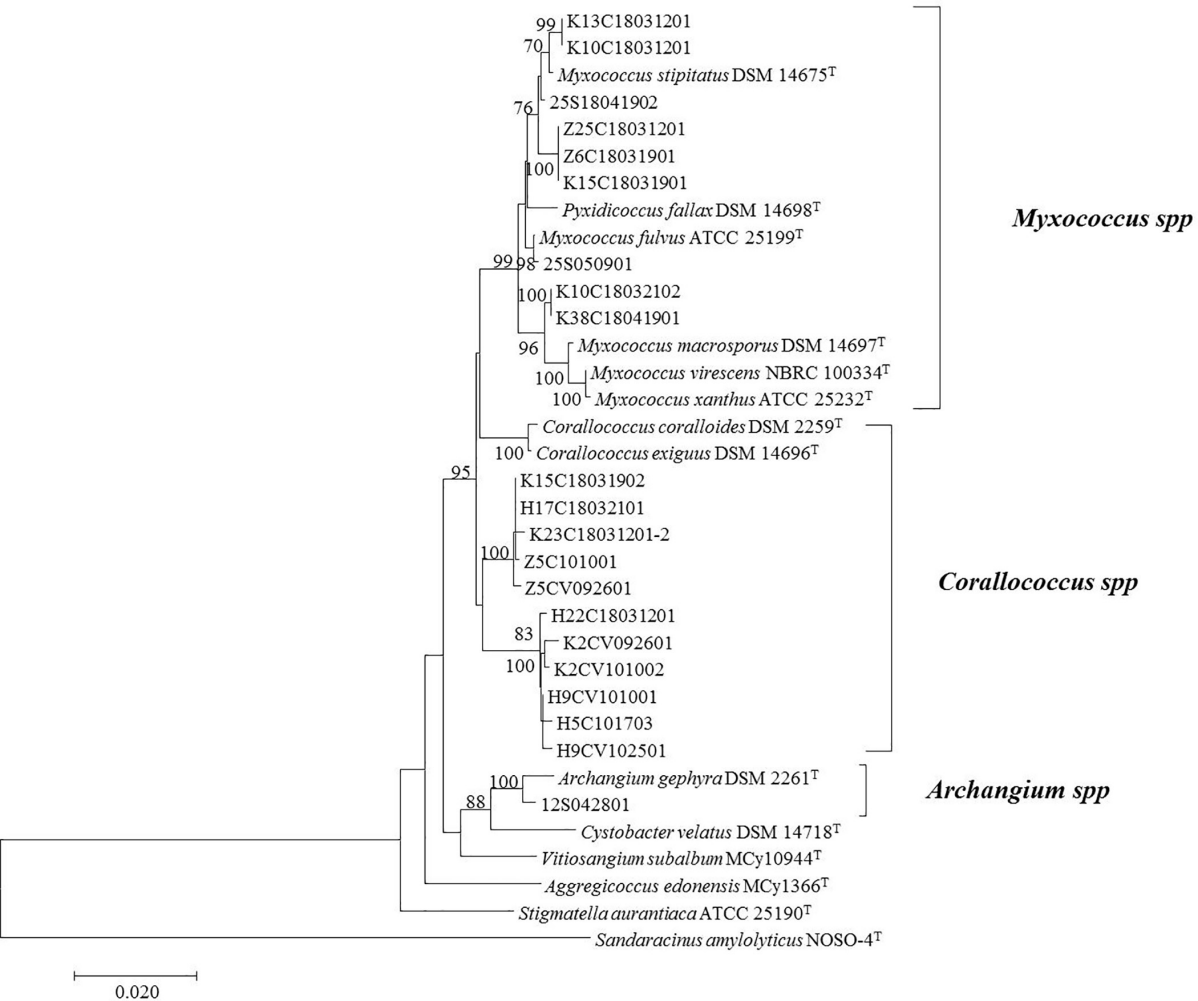
Neighbor-joining tree of cultures isolated from Dinghushan forest soil based on 16S rRNA gene sequences. Bootstrap values ˃70% are shown at branch points. Bar, 0.02 substitutions per nucleotide position.

A neighbor-joining tree was constructed based on the isolates’ 16S rRNA gene sequences, showing the strains grouped into 3 clusters with strong bootstrap support (Fig 2). Among these strains, 9 strains belong to *Myxococcus* and 11 strains belong to *Corallococcus*, exhibiting 97.7–99.8% homologies with known culture myxobacteria; 1 strain belongs to the genus *Archangium* in the family *Cystobacteriaceae*, showing 99.1% similarity to *Archangium gephyra*. Identities of myxobacteria obtained by 16S rRNA gene sequences analysis were listed in S1 Table.

Eleven strains of the genus *Corallococcus* showed 97.7–98.7% 16S rRNA gene sequence similarities with their most related species, which were lower than the threshold of 98.7% for species delineation [42], Strains H22C18031201, K2CV092601, K2CV101002, H9CV101001, H5C101703 and H9CV102501 revealed 99.3% 16S rRNA gene sequence similarities which possibly represent different strains of one potential novel species, and strains Z5C101001, K15C18031902, H17C18032101, K23C18031201-2 and Z5CV092601 showed 99.7% 16S rRNA gene sequence similarities which possibly represent different strains of the other potential novel species. We selected strains H22C18031201 and Z5C101001 as represent species for further analysis. According to comparative genome analysis, the ANI and dDDH values among strains H22C18031201, Z5C101001 and their closely relatives, showed 77.8–88.4% similarities and 20.9–34.7% similarities, respectively (Table 1). These values were much lower than the threshold of 95–96% and 70% for species delineation [42], supporting that strains H22C1803120 and Z5C101001 represent two potential novel species. In addition, strain 12S042801 showed one nucleotide difference of 16S rRNA gene sequence with *Archangium* sp. Cb G35. The ANI and dDDH values between *Archangium* sp. Cb G35 and *A*. *gephyra* were 92.3% and 45.9%, respectively. These values were also lower than the threshold of 95–96% and 70% for species delineation, indirect supporting strain 12S042801 may represent a potential novel species.

**Table 1:** Average nucleotide identity and digital DNA-DNA hybridization pairwise comparison of strains H22C18031201, Z5C101001 and their closely relatives.

	H22C18031201	Z5C101001	*C*. *coralloides*	*M*. *fulvus*	*M*. *macrosporus*	*M*. *stipitatus*	*M*. *virescens*	*M*. *xanthus*
1	**100**	79.3	79.1	78.1	78.8	77.8	78.3	78.0
2	22.1	**100**	88.4	78.5	79.0	78.4	78.6	78.5
3	21.7	34.7	**100**	78.6	79	78.1	78.4	78.4
4	21.1	23.6	21.2	**100**	80.9	83.6	80.4	80.1
5	21.4	21.7	21.5	23.3	**100**	80.3	90.6	90.4
6	20.9	21.0	20.9	26.5	22.8	**100**	80.1	79.8
7	21.1	21.4	21.3	22.9	40.5	22.5	**100**	97
8	21.1	21.4	24.6	22.7	40.1	22.3	73.1	**100**

The ANI values are shown on the upper side and the digital DDH values are shown on the lower side.

### Soil physicochemical properties

The pH of 9 soil samples was 3.6–4.5. The organic matter (OM) contents were 26.6–143.4 g/kg. Sample K30 had the lowest available nitrogen (AN) content of 88.7 mg/kg with the high available potassium (AK) content of 134 mg/kg. The available phosphorus (AP), ammonium (NH4^+^), nitrate (NO3^-^) and cation exchange capacity (CEC) contents did not differ significantly among 9 samples (S2 Table).

### Uncultured myxobacteria in Dinghushan forest soils with high-throughput sequencing method

**Fig 3:**
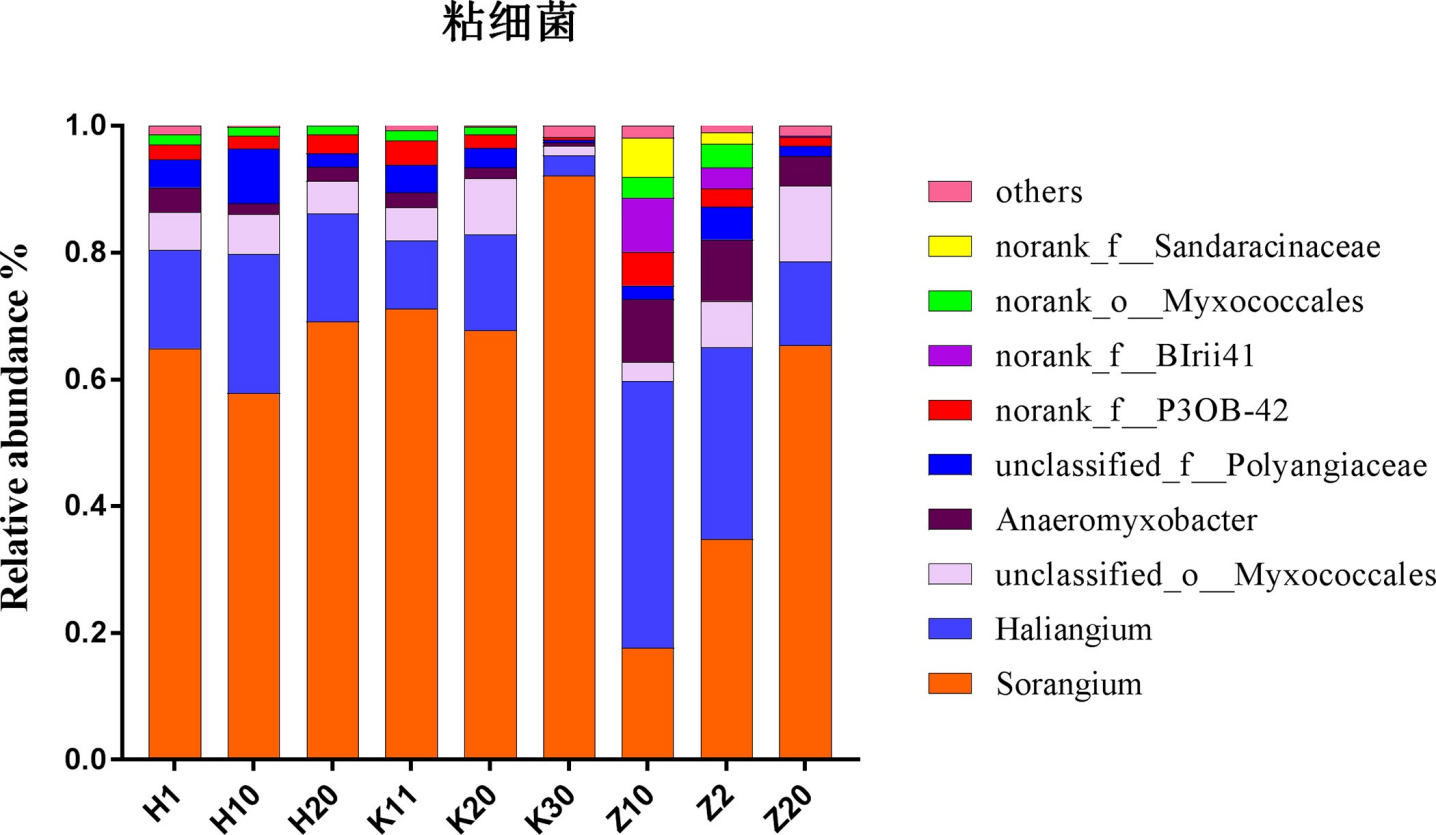
The myxobacterial community compositions on genus level of Dinghushan forest soils. Community compositions in soil samples were measured by relative abundance of 16S rRNA gene amplicon sequences.

We analyzed myxobacterial communities of 9 samples with high-throughput sequencing technique using the universal primers 338F and 806R. After merging, filtering and chimera removal, 46083–71424 16S rRNA gene amplicon sequences were finally recruited (S3 Table). The numbers of myxobacterial sequences ranged from 378 to 1091 in different soil samples. *Myxococcales* accounted for 0.9–2.2% of the total bacterial communities. *Sorangium* was the most dominant genus, accounting for 60.1% in *Myxococcales*, followed by the genus of *Haliangium*, accounting for 18.8%. *Anaeromyxobacter* was the fourth most dominant genus, accounting for 4.1%. In addition, *Sorangium* accounted for 94.7% in sample K30. All myxobacterial sequences grouped into 67 OTUs and the numbers ranged from 29 to 56 in different samples. The diversity and abundance of myxobacteria in soil samples exhibited significantly differences (Fig 3). The genera of g1_unclassified_*Sandaracinaceae* and g3_unclassified_BIrii41 were only present in samples Z2 and Z10 (Fig 3). These results showed an abundant myxobacterial community in Dinghushan acidic forest soils and the niche specificity of myxobacterial taxa. Confusingly, sequences of *Myxococcus* and *Corallococcus* that were isolated with culture-dependent technique were not obtained with culture-independent method in the same forest soil samples.

### Biotic and abiotic factors shaping myxobacterial community

To investigate the effects of environmental factors on myxobacterial community, we performed Spearman analysis of environmental factors and myxobacterial alpha-diversity and Mantel test of environmental factors and myxobacterial community structure. The results indicated that soil pH showed strong positive relationship with myxobacterial alpha-diversity (r = 0.817, *p*<0.01) and community structure (r = 0.5941, *p*<0.05). AK showed positive relationship with myxobacterial community structure (r = 0.4868, *p*<0.05), whereas AN, AP, OM, NH4^+^, NO3^-^ and CEC showed no relationship (Table 2). The alpha-diversity of bacteria (Chao and Shannon index) was significant positive correlated with alpha-diversity and community structure of myxobacteria (Table 2). These results all indicated that bacterial diversity and abiotic factors collaboratively shape myxobacterial community.

**Table 2:** The relationships between abiotic and biotic factors and myxobacterial alpha-diversity and community structure.

Environmental factors		Myxobacterial alpha-diversity		Myxobacterial community structure	
		r	*p*	r	*p*
	AN	0.545	0.113	0.144	0.257
	AP	0.327	0.391	-0.168	0.776
	**AK**	0.634	0.067	**0.489**	**0.035**
Abiotic factors	**pH**	**0.817**	**0.007**	**0.594**	**0.024**
	OM	0.426	0.253	0.084	0.283
	NH4^+^	0.257	0.504	-0.354	0.996
	NO3^-^	0.416	0.266	-0.303	0.933
	CEC	0.366	0.332	0.159	0.263
Biotic factors	Bacterial Chao	**0.753**	**0.019**	**0.314**	**0.032**
	Bacterial Shannon	**0.703**	**0.035**	0.398	0.097

The correlation between environmental factors and myxobacterial alpha-diversity was indicated by Spearman analysis. The correlation between environmental factors and myxobacterial community structure was revealed by Mantel test based on pairwise Bray-Curtis distance.

### Studying the relationships between myxobacteria and bacteria using co-occurrence network analysis

**Fig 4:**
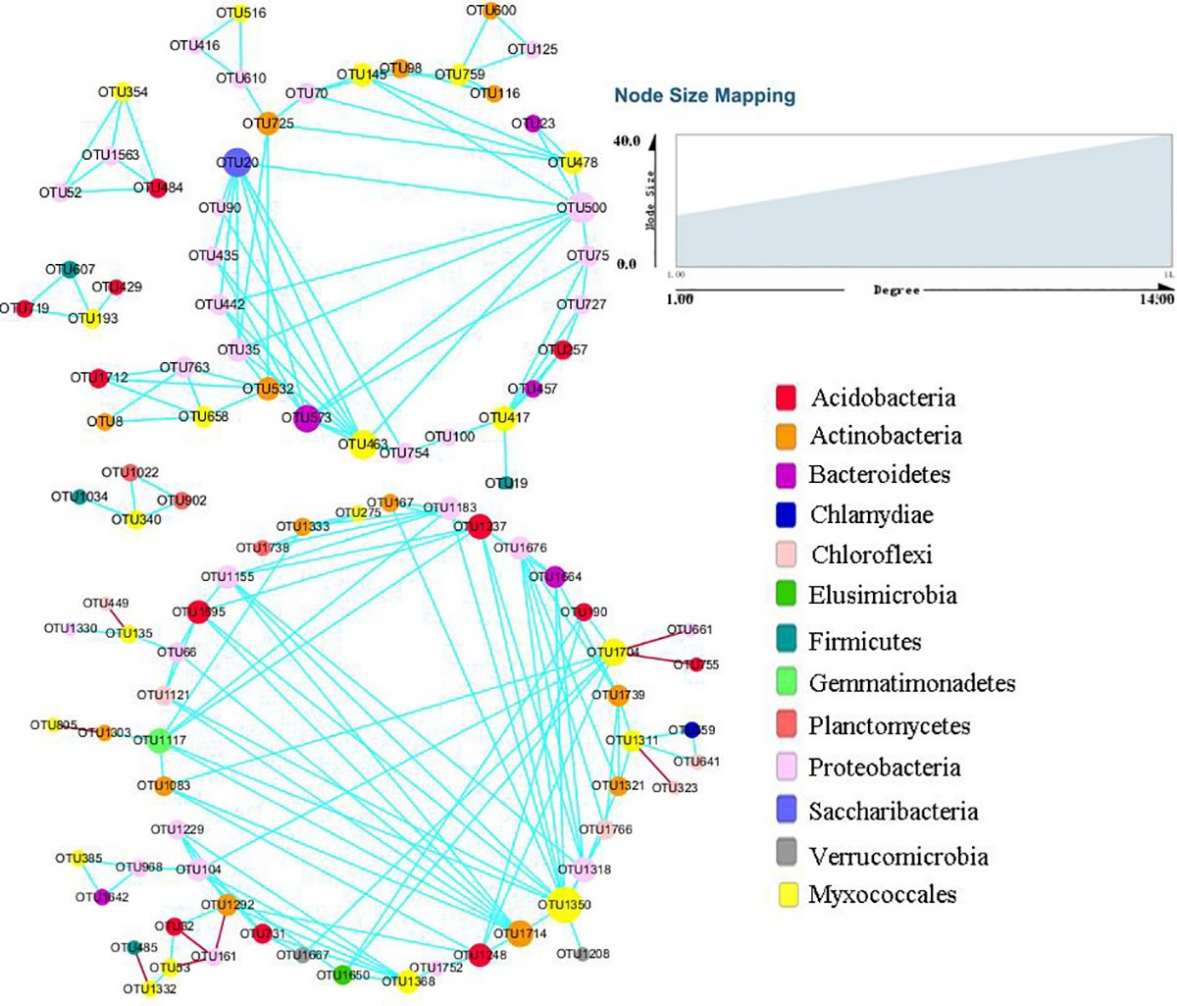
Sub-network of myxobacteria showed their direct interactions with other bacteria. Node represents individual OTU. The different colors signify the different phyla of bacteria. The yellow color represents *Myxococcales*. The blue solid line represents a positive correlation, and the red solid line signifies a negative correlation.

Co-occurrence networks were generated based on the OTU table to investigate the relationships between myxobacterial and other bacterial OTUs. Significant interactions were determined using a random matrix theory (RMT)-based on an online pipeline (http://ieg4.rccc.ou.edu/MENA). After filtering, a total of 20 OTUs from *Myxococcales* (yellow nodes) and 77 OTUs from other bacteria (other colors nodes) were contained, and most links were positive correlations (blue lines) among them (Fig 4). Myxobacteria have many links with other bacteria, especially *Proteobacteria*, probably indicating a broad range of bacteria as potential prey of myxobacteria. In particular, myxobacterial OTU1350, OTU463 and OTU1704 possessed 13, 8 and 8 links with other bacterial OTUs. Interestingly, in co-occurrence network, OTU457 representing strain *Dinghuibacter silviterrae* in the family of *Chitinophagaceae* isolated from Dinghushan forest soils [43] showed positive relationship with OTU417 representing uncultured *Haliangium* in the family of *Haliangiaceae*. In addition, OTU1664 representing unclassified *Chitinophagaceae* had also positive impact on OTU1350 representing P3OB-42 of myxobacteria.

## Discussion

Myxobacteria are one of the important sources for secondary metabolites aside from actinomycetes and fungi [44, 45]. To obtain these potential sources, there is a pressing need to screen the myxobacteria from different environment especially extreme and/or neglected habitats [46]. In this study, we investigated myxobacterial communities in Dinghushan acidic forest soils with pH 3.6–4.5 by using culture-dependent and -independent techniques, and analyzed the effect of biotic and abiotic factors on soil myxobacteria.

A total of 21 strains were isolated using standard cultivation methods. Almost all culturable strains were identified as the genera *Myxococcus* and *Corallococcus* (Fig 1). However, members of the two genera were not detected in the high-throughput sequencing analysis. This phenomenon has also been found in the study by Mohr *et al*. (2017). Combined with these analyses, we inferred that *Corallococcus*-strains were only present in forms of myxospores in-situ environments or that the vegetative cells/myxospores were strongly under-represented and therefore resisted the total genomic DNA extraction [11]. Similar to this study, *Myxococcus* and *Corallococcus* were frequently isolated from different habitats [11, 15], probably suggesting that they grow faster and easily form fruiting bodies. Intriguingly, three potential novel species were detected in all 21 culturable strains, indicating the high possibility to isolate new myxobacterial taxa from subtropical acidic forest soils. These potential novel species merit further identification in the future.

High-throughput sequencing revealed that the relative abundance of 16S rRNA amplicons of *Myxococcales* accounted for 0.9–2.2% of bacterial communities in Dinghushan acidic forest soils with pH of 3.6–4.5, in consistent with the result of Zhou *et al* [9], who revealed that the abundance of *Myxococcales* were among 0.4–4.5% in 103 various soil niches. Further, a total of 67 myxobacterial OTUs obtained from 9 soil samples exhibited an abundant myxobacterial community. Different soil samples exhibited significantly differences in the abundance and diversity of myxobacteria (Fig 3), which may relate to environmental factors. Correlation analysis revealed that soil pH was key positive correlated with myxobacterial community (Table 2), which were in accordance with previously studies [9, 11, 17]. Moreover, the sequences of *Myxococcus* and *Corallococcus* were detected in neutral environments and were not found in acidic habitats [9, 11, 47]. These may be due to the pH range for growth of the majority of myxobacteria is rather narrow. However, *Sorangium* in *Myxococcales* accounted for 60.1% in total samples and 94.7% in sample K30 (Fig 3). We inferred that pH range for growth of *Sorangium* is rather broad and acidic forest soils are potential habitats to obtain the genus of *Sorangium* which is frequently described as a rich source for new, biologically active natural products [20, 48]. Various myxobacterial species tolerant to different pH range, which will guide to isolate different myxobacterial taxa.

Bacterial diversity also showed a significantly positive relationship with myxobacterial community (Table 2). The network analysis further demonstrated that a large number of bacteria including two *Chitinophagaceae* OTUs were positive relationship with myxobacteria OTUs (Fig 4). Besides, four novel strains of the genus *Chitinophaga* [38–40] and one novel strain of the family *Chitinophagaceae* [41] were obtained during the isolation of myxobacteria. Our predation assays also indicated that myxobacteria could prey some strains of the genus *Chitinophaga*. Based on the above results, we speculated that some strains of the family *Chitinophagaceae* are the potential food sources of myxobacteria.

Taken together, subtropical acidic forest soils contain abundance myxobacterial community, especially the genera of *Sorangium* and *Haliangium*. Moreover, soil pH and bacterial diversity have significantly positive relationship with myxobacterial community. Therefore, trying different pH media and various bacteria such as *Chitinophagaceae* as preys to isolate myxobacteria maybe contribute to obtain more novel groups.

## Supporting information

S1 Table Identities of myxobacteria obtained by 16S rRNA gene sequence blast. (DOCX)

S2 Table Soil chemical characteristics of Dinghushan forest soil. OM, organic matter; AP, available phosphorus; AN, available nitrogen; AK, available potassium; NH_4_^+^, ammonium; NO3^-^, nitrate; CEC, cation exchange capacity.
(DOCX)

S3 Table Observed valid sequences and myxobacterial OTUs at different samples. (DOCX)
