# Supplementary material for: Culture-dependent and -independent methods revealed an abundant myxobacterial community shaped by other bacteria and pH in Dinghushan acidic soils
Source: PLoS One. 2020 Sep 14;15(9):e0238769. doi: 10.1371/journal.pone.0238769 (PMC7489521; doi:10.1371/journal.pone.0238769)
Supplement: S1 Table — (DOCX) [file pone.0238769.s001.docx]

**S1 Table.** **Identities of myxobacteria obtained by 16S rRNA gene sequence blast.**

| Code | Cluster representative | Closest type strain | Similarity（%） |
| --- | --- | --- | --- |
| 1 | K23C18031201-2 | *Corallococcus exiguous* DSM 14696^T^ | 97.8 |
| 2 | H17C18031201 | *Corallococcus exiguous* DSM 14696^T^ | 98.6 |
| 3 | K15C18031902 | *Corallococcus exiguous* DSM 14696^T^ | 98.0 |
| 4 | Z5C101001 | *Corallococcus exiguous* DSM 14696^T^ | 98.7 |
| 5 | Z5CV092601 | *Corallococcus exiguous* DSM 14696^T^ | 98.6 |
| 6 | H22C18031201 | *Corallococcus coralloides* DSM 2259^T^ | 98.1 |
| 7 | K2CV101002 | *Corallococcus coralloides* DSM 2259^T^ | 97.8 |
| 8 | K2CV092601 | *Corallococcus coralloides* DSM 2259^T^ | 97.7 |
| 9 | H9CV101001 | *Corallococcus coralloides* DSM 2259^T^ | 98.1 |
| 10 | H5C101703 | *Corallococcus coralloides* DSM 2259^T^ | 97.8 |
| 11 | H9CV102501 | *Corallococcus coralloides* DSM 2259^T^ | 97.8 |
| 12 | K15C18031901 | *Myxococcus stipitatus* DSM 14675^T^ | 99.8 |
| 13 | Z6C18031901 | *Myxococcus stipitatus* DSM 14675^T^ | 99.6 |
| 14 | Z25C18031201 | *Myxococcus stipitatus* DSM 14675^T^ | 99.7 |
| 15 | K10C18032101 | *Myxococcus stipitatus* DSM 14675^T^ | 99.7 |
| 16 | K13C18031201 | *Myxococcus stipitatus* DSM 14675^T^ | 99.7 |
| 17 | 25S18041901 | *Myxococcus stipitatus* DSM 14675^T^ | 99.6 |
| 18 | K10C18032102 | *Myxococcus fulvus* DSM 16525^T^ | 99.5 |
| 19 | 25S050901 | *Myxococcus fulvus* DSM 16525^T^ | 99.1 |
| 20 | K38C18041901 | *Myxococcus xanthus* DSM 16526^T^ | 99.3 |
| 21 | 12S042801 | *Archangium gephyra* DSM 2261^T^ | 99.1 |
