# Supplementary material for: Culture-dependent and -independent methods revealed an abundant myxobacterial community shaped by other bacteria and pH in Dinghushan acidic soils
Source: PLoS One. 2020 Sep 14;15(9):e0238769. doi: 10.1371/journal.pone.0238769 (PMC7489521; doi:10.1371/journal.pone.0238769)
Supplement: S2 Table — OM, organic matter; AP, available phosphorus; AN, available nitrogen; AK, available potassium; NH4+, ammonium; NO3-, nitrate; CEC, cation exchange capacity. (DOCX) [file pone.0238769.s002.docx]

**S2 Table. Soil chemical characteristics of Dinghushan forest soil.**

| Sample | pH _(water)_ | OM (g/kg) | AP (mg/kg) | AN (mg/kg) | AK (mg/kg) | NH_4_^+^ (mg/kg) | NO_3_^-^ (mg/kg) | CEC (cmol/kg) |
| --- | --- | --- | --- | --- | --- | --- | --- | --- |
| K11 | 3.9 | 52.9 | 0.16 | 190.9 | 87 | 24.9 | 7.8 | 19.2 |
| K20 | 3.7 | 113.8 | 0.13 | 232.4 | 51 | 17.6 | 12.4 | 26 |
| K30 | 4.0 | 26.6 | 0.06 | 88.7 | 134 | 26.4 | 5.2 | 12.6 |
| Z2 | 4.4 | 135 | 0.22 | 347.6 | 85 | 29.5 | 14 | 32.8 |
| Z10 | 4.5 | 143.4 | 0.12 | 292.6 | 139 | 19.7 | 9.8 | 25.5 |
| Z20 | 4.0 | 29.6 | 0.05 | 69.9 | 27 | 14.5 | 1.5 | 19.8 |
| H1 | 3.9 | 121.6 | 0.15 | 239.5 | 62 | 41.4 | 31.8 | 20.5 |
| H10 | 3.6 | 141.1 | 0.18 | 265.4 | 58 | 36.3 | 11 | 32.1 |
| H20 | 3.6 | 131.9 | 0.07 | 236.9 | 45 | 17.1 | 3.6 | 24.4 |

OM, organic matter; AP, available phosphorus; AN, available nitrogen; AK, available potassium; NH_4_^+^, ammonium; NO3^-^, nitrate; CEC, cation exchange capacity.
