# Supplementary material for: Culture-dependent and -independent methods revealed an abundant myxobacterial community shaped by other bacteria and pH in Dinghushan acidic soils
Source: PLoS One. 2020 Sep 14;15(9):e0238769. doi: 10.1371/journal.pone.0238769 (PMC7489521; doi:10.1371/journal.pone.0238769)
Supplement: S3 Table — (DOCX) [file pone.0238769.s003.docx]

**S3 Table.** **Observed valid sequences and myxobacterial OTUs at different samples**

| Sample | Even sequence number | Base number | Average length | Myxobacterial OTU | Coverge |
| --- | --- | --- | --- | --- | --- |
| H1 | 51357 | 22210651 | 432.48 | 48 | 0.99 |
| H10 | 64792 | 27976287 | 431.79 | 41 | 0.99 |
| H20 | 69811 | 30140005 | 431.74 | 34 | 0.99 |
| K11 | 53872 | 23368749 | 433.78 | 38 | 0.99 |
| K20 | 58110 | 25142133 | 432.66 | 29 | 0.99 |
| K30 | 71387 | 30879773 | 432.57 | 30 | 0.99 |
| Z10 | 71424 | 31060514 | 434.88 | 55 | 0.99 |
| Z2 | 53091 | 22999330 | 433.21 | 56 | 0.98 |
| Z20 | 46083 | 20058474 | 435.27 | 37 | 0.99 |
